# Network meta-analysis of the efficacy of pharmacological treatments for post-stroke cognitive impairment and vascular cognitive impairment

**DOI:** 10.3389/fneur.2025.1683496

**Published:** 2025-12-01

**Authors:** Wenting Li, Xinyu Liu, Cong Gao, Wenbo Li, Xiaoling Liao

**Affiliations:** Department of Neurology, Tiantan Hospital, Capital Medical University, Beijing, China

**Keywords:** pharmacological treatments, memantine, cholinesterase inhibitors, traditional Chinese medicine, post-stroke cognitive impairment

## Abstract

**Background:**

Based on recent reviews, vascular cognitive impairment (VCI) encompasses a spectrum of cognitive deficits caused by cerebrovascular disease and its risk factors, ranging from mild cognitive impairment to dementia, and often coexists with neurodegenerative conditions like Alzheimer’s disease. VCI is categorized into four clinical-imaging subtypes, including post-stroke cognitive impairment (PSCI)—a common stroke complication and major VCI subtype. Current guidelines recommend cholinesterase inhibitors and NMDA receptor antagonists as first-line treatments for VCI, with expert consensus supporting donepezil and rivastigmine for PSCI. However, existing evidence primarily derives from placebo-controlled or head-to-head drug comparisons, lacking comprehensive evaluations of multiple cognitive enhancers. This study aims to systematically assess the efficacy and safety of cognitive-enhancing drugs in VCI, with a focused analysis on PSCI, to better inform clinical decision-making and improve patient outcomes.

**Methods:**

We systematically searched four databases using predefined search strategies. Eligible studies were selected based on predetermined criteria. The included studies were analyzed with StataSE 16.0, RevMan 5.3, and Grade software to compare the efficacy and safety of cognitive-enhancing drugs to identify the optimal treatment for VCI and PSCI.

**Results:**

Sixteen studies (5,599 participants) were included. In terms of cognitive outcomes, sailuotong was superior to placebo on the Alzheimer’s Disease Assessment Scale-Cognitive Subscale (ADAS-cog) (MD = −3.00, 95% CI: −4.50, −1.50) and ranked best (SUCRA 88.5%). Memantine was most effective on the Mini-Mental State Examination (MMSE) (MD = 1.23, 95% CI: 0.23–2.23; SUCRA 80.8%). For the secondary outcome, the MoCA assessment showed that *Ginkgo biloba* extract significantly improved Montreal Cognitive Assessment (MoCA) scores compared to placebo (MD = 1.29, 95% CI: 1.24, 1.35). Regarding safety, donepezil significantly increased the risk of overall adverse events compared to placebo (OR: 1.57; 95% CI: 1.19–2.06).

**Conclusion:**

Our network meta-analysis suggests that memantine might have the best effect for PSCI, with sailuotong potentially serving as a secondary option. However, these estimates are based on a small randomized controlled trial and a sparse network. Therefore, the current evidence is limited, highlighting the need for more high-quality studies to robustly validate the therapeutic potential of these interventions for VCI and PSCI.

**Systematic review registration:**

https://www.crd.york.ac.uk/PROSPERO/, identifier in PROSPERO (CRD420250627957).

## Introduction

According to the latest review on cognitive impairment (VCI), it refers to cognitive dysfunction primarily caused by cerebrovascular diseases and their risk factors. This condition encompasses the entire spectrum from mild cognitive impairment (MCI) to dementia and can coexist with neurodegenerative diseases such as Alzheimer’s disease (AD) (1). Based on its clinical features and imaging manifestations, VCI can be classified into four subtypes (2, 3): (1) post-stroke cognitive impairment (PSCI), (2) subcortical ischemic vascular cognitive impairment, (3) multi-infarct cognitive impairment, (4) mixed cognitive impairment, among these, PSCI is both a key subtype of VCI and a prevalent complication following stroke. According to the review published in the 2025 World Stroke Organization Global Stroke Fact Sheet, stroke remains the second leading cause of death worldwide (approximately 7 million people) and the third leading cause of death and disability among non-communicable diseases (3). A systematic review that included 23 studies published between 1995 and 2017 found a pooled prevalence of PSCI without dementia in the first year after stroke of 38% (95% CI: 32, 43%) (4). To improve the outcomes of patients with cerebrovascular disease and reduce the incidence of VCI and PSCI, the appropriate management of these patients has become particularly important.

Currently available cognitive-enhancing drugs are primarily indicated for AD, and none are officially approved for VCI or PSCI. Some studies have reported modest therapeutic effects of these agents in PSCI patients. According to the latest VCI management guidelines (3), acetylcholinesterase inhibitors and NMDA receptor antagonists are recommended as first-line treatments. Meanwhile, a Chinese expert consensus on PSCI management indicates that the cholinesterase inhibitors donepezil and rivastigmine can be used as first-line therapies to improve cognitive function and activities of daily living (5). The management perspectives on PSCI from the American Heart Association/American Stroke Association 2023 largely focus on the prevention of risk factors related to cerebral infarction (6).

There are currently numerous cognition-enhancing medications available for patients with VCI, like acetylcholinesterase inhibitors, memantine, butylphthalide, ginkgo biloba extract, and traditional Chinese medicines. But most existing reviews or meta-analyses on PSCI pharmacotherapy focus on efficacy comparisons between traditional medications and placebos or between two individual cognitive-enhancing drugs (7, 8), with a dearth of meta-analyses evaluating multiple cognitive-enhancing agents. To assist clinicians and patients in better understanding cognitive-enhancing medications and improving VCI prognosis, this study summarizes pharmacological research and compares their effectiveness and adverse events for VCI patients, with a separate analysis dedicated to PSCI as an important subtype.

## Materials and methods

This study followed the Preferred Reporting Items for Systematic Reviews and Meta-Analyses (PRISMA) guidelines (9), and the study protocol has been registered on the PROSPERO (Registration number: CRD420250627957).

### Search strategy

This study conducted searches across four databases: PubMed, Cochrane, Web of Science and, Embase. The search strategy included MeSH terms, subject headings, and keywords such as “stroke,” “brain Ischemia,” “cerebral hemorrhage,” “transient ischemic attack,” “subarachnoid hemorrhage,” “cognitive dysfunction,” “cholinesterase inhibitors,” “galantamine,” “nimodipine,” “cytidine diphosphate choline,” “pentoxifylline,” “traditional Chinese medicine,” “memantine,” “rivastigmine” and “ginkgo biloba extract. The specific PubMed search method is detailed in Table 1, and the search strategies for other databases were all adapted from the PubMed search strategy.

**Table 1:** The search strategies.

Pubmed	
#1	((((((((((((((((((((((((((((((((((((((((((((((((((((((“Stroke”[MeSH Terms]) OR (“Brain Ischemia”[MeSH Terms])) OR (“Cerebral Hemorrhage”[MeSH Terms])) OR (“Ischemic Attack, Transient”[MeSH Terms])) OR (“Subarachnoid Hemorrhage”[MeSH Terms])) OR (Stroke[Title/Abstract])) OR (Cerebrovascular Accident[Title/Abstract])) OR (Brain Vascular Accident[Title/Abstract])) OR (Apoplexy[Title/Abstract])) OR (apoplex[Title/Abstract])) OR (“brain attack”[Title/Abstract])) OR (“brain insult”[Title/Abstract])) OR (“ischemic seizure”[Title/Abstract])) OR (“Brain Infarct”[Title/Abstract])) OR (“Cerebral Infarct”[Title/Abstract])) OR (“Brain Stem Infarct”[Title/Abstract])) OR (“Subcortical Infarction”[Title/Abstract])) OR (“Brain Venous Infarction”[Title/Abstract])) OR (“Cerebral Artery Stroke”[Title/Abstract])) OR (“Cerebral Artery Infarction”[Title/Abstract])) OR (“Cerebral Circulation Infarction”[Title/Abstract])) OR (“Circulation Brain Infarction”[Title/Abstract])) OR (“Choroidal Artery Infarction”[Title/Abstract])) OR (CVA[Title/Abstract])) OR (CVAs[Title/Abstract])) OR (“Brain Ischemia”[Title/Abstract])) OR (“Cerebral Ischemia”[Title/Abstract])) OR (“Brain Hypoxia Ischemia”[Title/Abstract])) OR (“Cerebral Anoxia Ischemia”[Title/Abstract])) OR (“Cerebral Hemorrhage”[Title/Abstract])) OR (“Cerebral Brain Hemorrhage”[Title/Abstract])) OR (“Cerebral Parenchymal Hemorrhage”[Title/Abstract])) OR (“Intracerebral Hemorrhage”[Title/Abstract])) OR (“Basal Ganglia Hemorrhage”[Title/Abstract])) OR (“Subarachnoid Hemorrhage”[Title/Abstract])) OR (“Cerebral Hypertensive Hemorrhage”[Title/Abstract])) OR (“Brain TIA”[Title/Abstract])) OR (“TIA, Brain”[Title/Abstract])) OR (TIA (Transient Ischemic Attack[Title/Abstract]))) OR (TIAs (Transient Ischemic Attack[Title/Abstract]))) OR (“Transient Ischemic Attack”[Title/Abstract])) OR (“Attacks, Transient Ischemic”[Title/Abstract])) OR (“Ischemic Attacks, Transient”[Title/Abstract])) OR (“Transient Ischemic Attacks”[Title/Abstract])) OR (“Cerebral Ischemia, Transient”[Title/Abstract])) OR (“Transient Ischemic Attack, Brainstem”[Title/Abstract])) OR (“Hemorrhage, Subarachnoid”[Title/Abstract])) OR (“Hemorrhages, Subarachnoid”[Title/Abstract])) OR (“Subarachnoid Hemorrhages”[Title/Abstract])) OR (SAH (Subarachnoid Hemorrhage[Title/Abstract]))) OR (SAHs (Subarachnoid Hemorrhage[Title/Abstract]))) OR (“Aneurysmal Subarachnoid Hemorrhage”[Title/Abstract])) OR (“Aneurysmal Subarachnoid Hemorrhages”[Title/Abstract])) OR (“Hemorrhage, Aneurysmal Subarachnoid”[Title/Abstract])) OR (“Subarachnoid Hemorrhage, Spontaneous”[Title/Abstract])
#2	(((((((((((((((((((((“cognitive dysfunction”[MeSH Terms]) OR (“Cognition Disorders”[MeSH Terms])) OR (“Dementia, Vascular”[MeSH Terms])) OR (“Cognitive Decline”[Title/Abstract])) OR (amnestic[Title/Abstract])) OR (“cognition disorder”[Title/Abstract])) OR (“cognitive defect”[Title/Abstract])) OR (“cognitive deficit”[Title/Abstract])) OR (“cognitive disability”[Title/Abstract])) OR (“cognitive disorder”[Title/Abstract])) OR (“cognitive dysfunction”[Title/Abstract])) OR (“cognitive impairment”[Title/Abstract])) OR (delirium[Title/Abstract])) OR (“mental deterioration”[Title/Abstract])) OR (“overinclusion”[Title/Abstract])) OR (“response interference”[Title/Abstract])) OR (“Vascular Dementia”[Title/Abstract])) OR (“Mild Neurocognitive Disorder”[Title/Abstract])) OR (“Multi-Infarct Dementia”[Title/Abstract])) OR (“Lacunar Dementia”[Title/Abstract])) OR (“Arteriosclerotic Dementia”[Title/Abstract])) OR (“Chronic Progressive Subcortical Encephalopathy”[Title/Abstract])
#3	((((((“cholinesterase inhibitors”[MeSH Terms]) OR (galantamine[MeSH Terms])) OR (Nimodipine[MeSH Terms])) OR (“Cytidine Diphosphate Choline”[MeSH Terms])) OR (Pentoxifylline[MeSH Terms])) OR (“Medicine, Chinese Traditional”[MeSH Terms])) OR (Memantine[MeSH Terms])
#4	(((((((((((((((((((((((((((((((((((((GV-971[Title/Abstract]) OR (donepezil[Title/Abstract])) OR (rivastigmine[Title/Abstract])) OR (Oxiracetam[Title/Abstract])) OR (“*Ginkgo biloba* extract”[Title/Abstract])) OR (“Ginkgo leaf extract”[Title/Abstract])) OR (Tebokan[Title/Abstract])) OR (Tebonin[Title/Abstract])) OR (“EGb 761”[Title/Abstract])) OR (Nimotop[Title/Abstract])) OR (“Bay e 9,736”[Title/Abstract])) OR (Admon[Title/Abstract])) OR (Brainal[Title/Abstract])) OR (N-butylphthalide[Title/Abstract])) OR (Butylphthalide[Title/Abstract])) OR (DL-3-n-butylphthalide[Title/Abstract])) OR ((S)-(−)-3-butylphthalide[Title/Abstract])) OR (“Inhibitors, Cholinesterase”[Title/Abstract])) OR (Anti-Cholinesterase[Title/Abstract])) OR (Anti Cholinesterase[Title/Abstract])) OR (“Anticholinesterase Agent”[Title/Abstract])) OR (Oxiracetam[Title/Abstract])) OR (“Cytidine Diphosphate Choline”[Title/Abstract])) OR (“Choline, Cytidine Diphosphate”[Title/Abstract])) OR (“Diphosphate Choline, Cytidine”[Title/Abstract])) OR (Citicoline[Title/Abstract])) OR (Oxpentifylline[Title/Abstract])) OR (Trental[Title/Abstract])) OR (Agapurin[Title/Abstract])) OR (Torental[Title/Abstract])) OR (BL-191[Title/Abstract])) OR (“Traditional Medicine, Chinese”[Title/Abstract])) OR (“Chinese Traditional Medicine”[Title/Abstract])) OR (“Traditional Tongue Diagnosis”[Title/Abstract])) OR (“1,3-Dimethyl-5-aminoadamantane”[Title/Abstract])) OR (“1-Amino-3,5-dimethyladamantane”[Title/Abstract])) OR (Memantin[Title/Abstract])) OR (D-145[Title/Abstract])
#5	(((“randomized controlled trial”[Title/Abstract]) OR (randomized[Title/Abstract])) OR (placebo[Title/Abstract]))
#6	#1 AND #2 AND #3 AND #4 AND #5

### Eligibility and exclusion criteria

Eligibility Criteria: (1) patients: patients diagnosed with cerebrovascular disease and subsequent cognitive impairment, confirmed by cognitive scale scores below the lower limit of normal. No significant cognitive dysfunction prior to cerebrovascular disease. (2) Interventions: pharmacological treatments with proven efficacy in improving cognition, including but not limited to: acetylcholinesterase inhibitors (e.g., donepezil, rivastigmine, galantamine), memantine, butylphthalide (DL-3-n-butylphthalide), ginkgo biloba extract, and traditional Chinese medicine. (3) Comparators: placebo or other active cognitive-enhancing drugs. (4) Outcomes: primary outcomes assessed by the following scales: Mini-Mental State Examination (MMSE), Alzheimer’s Disease Assessment Scale-Cognitive Subscale (ADAS-cog), Montreal Cognitive Assessment (MoCA) scores as a secondary outcome. (5) Study design: randomized controlled trials (RCTs) only. Exclusion criteria: (1) Patients without VCI or PSCI. (2) Interventions involving non-pharmacological therapies. (3) Duplicate publications. (4) Studies published in languages other than English and Chinese. (5) Full-text unavailable. (6) Studies with unclear/missing patient, intervention, comparator, outcome, and study (PICOS) criteria. (7) Non-pharmacological interventions.

### Data extraction

The retrieved literature from our search strategy was imported into EndNote X9, where duplicate records were identified and removed using the software’s deduplication function. Two graduate researchers (A and B) in this field independently screened all studies by reviewing titles and abstracts according to the predefined inclusion and exclusion criteria.

Studies that passed this initial screening phase had their full texts obtained for further evaluation. The same researchers then conducted full-text reviews using the same eligibility criteria. During the data extraction process, any disagreements between reviewers A and B were first addressed through discussion. If consensus could not be reached through consultation, a senior expert in the field was consulted to make the final determination regarding the inclusion of disputed studies.

### Statistical analysis

The retrieved literature was imported into RevMan 5.3 software and grouped according to cognitive assessment scales. The effect size was determined based on the mean change (mean_change_) and change in standard deviation (SD_change_). When only the standard error (SE) was reported, SD was calculated as SE × √N (N = sample size). For studies with multiple subgroups, the following formula was used: SD=$\surd \frac{\left(N1-1\right)\mathrm{SD}12+\left(N2-1\right)\mathrm{SD}22+\frac{N1N2}{N1+N2}\left(M12+M22-2M1M2\right))}{N1+N2-1}$,If mean_change_ or SD_change_ was not available, the following formula was applied: mean_change_ = mean_final_-mean_baseline_ SD_change_=√SD_final_^2^ + SD_baseline_^2^-2r × SD_final_ × SD_baseline_, (*r* = 0.5). Pairwise meta-analyses were performed for studies with the same interventions to enable side-by-side comparisons, generating forest plots and calculating mean differences (MD) with 95% confidence intervals (CI). Heterogeneity was assessed using I^2^ statistics and *p*-values, with a fixed-effect model selected when I^2^ < 50% and *p* > 0.1; otherwise, a random-effects model was used.

For network meta-analysis, StataSE 16.0 software with the network package was employed. Network plots were generated based on cognitive assessment scales, where node sizes represented intervention sample sizes and connection widths indicated the number of pairwise comparisons. Global inconsistency tests were conducted to evaluate overall network consistency. For networks containing closed loops, local inconsistency was assessed using the node-splitting method (*p* > 0.05 indicating no significant inconsistency) and loop inconsistency evaluation (where confidence intervals excluding 0 or large IF values suggested substantial differences between direct and indirect comparisons). A cumulative probability ranking curve was created to quantify the effectiveness of different interventions, with higher SUCRA values indicating better efficacy. League tables were generated to compare effect sizes across interventions.

The GRADE framework was used to rate the quality of evidence from the network meta-analysis, considering risk of bias, inconsistency, indirectness, imprecision, and publication bias. Risk of bias was assessed through the risk of bias graph and the risk of bias summary generated by RevMan software. Inconsistency was evaluated by comparing confidence interval ranges in forest plots created using StataSE 16 software. Indirectness was determined based on whether indirect comparisons existed in the literature. Imprecision was assessed through forest plots in RevMan 5.3, where treatment effects with 95% confidence intervals excluding 0 and sample sizes >400 were considered precise (10); otherwise, the rating was downgraded. Publication bias was analyzed using funnel plots in StataSE 16.0, with asymmetry indicating potential bias. Evidence upgrading was not applied as all included studies were randomized controlled trials.

## Results

### Literature selection

**Figure 1:**
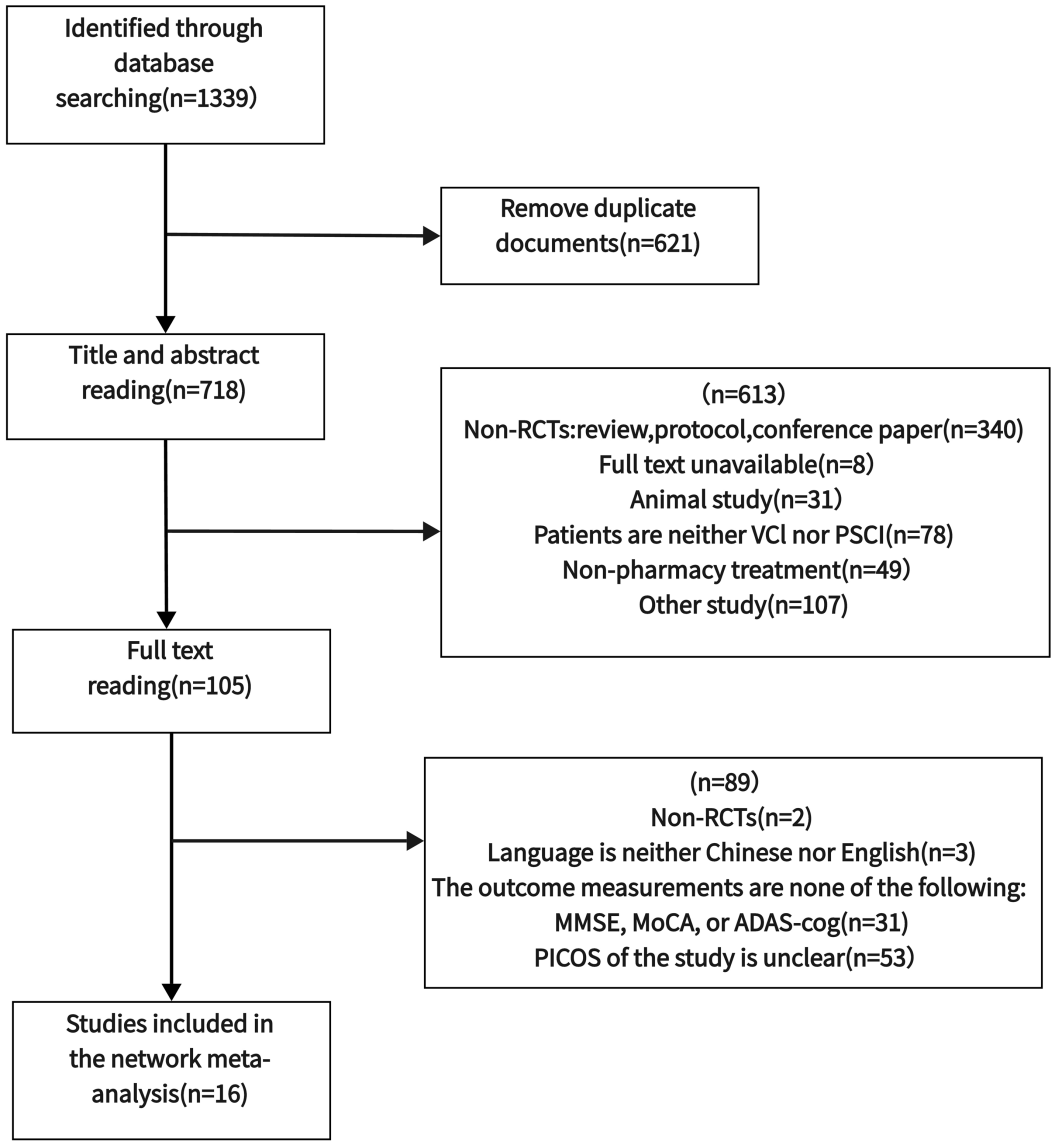
Flow diagram of eligible studies selection process. PICOS, Patient, intervention, compare, outcome, study; n, number of publications; VCI, vascular cognitive impairment; PSCI, post-stroke cognitive impairment.

The initial search retrieved 1,339 articles from four databases (PubMed, Cochrane, Web of Science, and Embase). After importing into EndNote X9 and removing 621 duplicates, we screened titles and abstracts of 718 articles, excluding 613 articles (340 non-RCTs, 8 unavailable full texts, 31 animal studies, 78 studies involving patients without VCI or PSCI, 49 non-pharmacological intervention studies, and 107 irrelevant studies). Full-text assessment of the remaining 105 articles led to further exclusion of 89 articles (2 non-RCTs, 3 non-English/Chinese publications, 31 studies not using MoCA/MMSE/ADAS-cog scales, 53 studies with unclear PICOS criteria). Finally, 16 eligible studies were included in the network meta-analysis, with the detailed selection process shown in Figure 1.

### Study characteristics

A total of 16 studies meeting the inclusion criteria were included, involving 5,599 participants. Of these, 6 studies with 1,309 participants focused on PSCI, while the remaining 10 studies, involving 4,290 participants, investigated VCI. The interventions included: galantamine (2 studies), donepezil (4 studies), rivastigmine (1 study), nimodipine (5 studies), ginkgo biloba extract (2 studies), and memantine (1 study), while sailuotong was examined in only one study. The treatment duration across these 16 studies ranged from 4 weeks to 62 weeks. All 6 studies on PSCI provided the timing of drug intervention, which ranged from 7 days to over 3 months. 1 study provided baseline scores for MMSE, ADAS-cog, and MoCA, 6 studies provided baseline scores for both MMSE and ADAS-cog, 1 study provided baseline scores for both MMSE and MoCA, 6 studies reported only MMSE baseline scores, 1 study reported only MoCA baseline scores, and another study failed to provide baseline scores for the above cognitive scales. Detailed characteristics of the 16 included studies are presented in Table 2 (VCI) and Table 3 (PSCI).

**Table 2:** Main characteristics of the eligible VCI studies.

Study	Treatment	Number of patients	Male	Age (Mean)	MMSE (Mean±SD)	ADAS-cog (Mean±SD)	Duration
Auchus 2007 (28)	placebo	390	256	72.2	20.2 ± 3.9	22.5 ± 9.5	26w
	Galantamine	396	247	72.3	20.3 ± 3.9	22.9 ± 9.5	
Román 2005 (24)	placebo	392	220	74.3	21.6 ± 4.2	22.3 ± 11.1	24w
	donepezil	827	482	74.51	21.5 ± 4.1	23.15 ± 11.54	
Black 2003 (25)	placebo	199	115	74.2	21.7 ± 4.23	20.1 ± 9.87	24w
	donepezil	404	218	73.8	21.85 ± 4.26	21.05 ± 10.65	
Liu 2022 (19)	placebo	30	10	74.1			16w
	sailuotong	32	12	75			
Mok 2007 (20)	placebo	20	9	74.1	13.4 ± 5.9		26w
	rivastigmine	20	7	75.7	13 ± 4.2		
Orgogozo 2002 (15)	placebo	141	80	76.1	16.9 ± 2.44	21.5 ± 8.71	28w
	memantine	147	72	76.6	16.9 ± 2.6	20.6 ± 9.55	
Pantoni 2000 (27)	placebo	126	58	74.8	21.4 ± 4.24		26w
	nimodipine	125	61	73.7	21.24 ± 4.07		
Pantoni 2005 (26)	placebo	109	67	75.4	20.5 ± 3.2		52w
	nimodipine	121	70	75.2	20 ± 3		
Small 2003 (29)	placebo	70	38	73.4	20.3 ± 3.35	23.5 ± 10.38	6 m
	galantamine	125	72	73.8	20.9 ± 3.24	21.5 ± 8.72	
Wilkinson 2003 (30)	placebo	193	105	74.4	18.8 ± 9.72	22.2 ± 4.17	24w
	donepezil	423	264	75.21	20.7 ± 10.17	21.65 ± 4.36	

**Table 3:** Main characteristics of the eligible PSCI studies.

Study	Treatment	Number of patients	Male	Age (Mean)	MMSE (Mean ± SD)	ADAS-cog (Mean±SD)	MoCA (Mean ± SD)	Duration	Treatment timepoint
Chang 2011 (23)	placebo	4	2	55	24.8 ± 1			4w	>3 m
	donepezil	6	4	55.5	24.2 ± 1.2				
Li 2017 (31)	placebo	165	69	63.3	22.6 ± 6.29		18.72 ± 6.94	6 m	<7d
	ginkgo biloba extract	177	57	64.5	22.6 ± 5.99		18.79 ± 6.79		
Moretti 2008 (32)	rivastigmine	50	24	73.2	20.7 ± 2.0			24w	3 m
	nimodipine	50	20	72.5	20.0 ± 2.1				
Sze 1998 (22)	placebo	42	25	71.07	24.52 ± 6.22			12w	7–14d
	nimodipine	44	28	70.25	25.36 ± 4.9				
Zheng, H 2019 (21)	placebo	291	212	60.1	26.8 ± 2.2	9.55 ± 4.2	20.8 ± 3.3	6 m	7d
	nimodipine	287	222	60.9	26.8 ± 2.3	9.28 ± 3.6	20.84 ± 3.7		
Cui 2023 (33)	placebo	96	76	64.1		23.47 ± 4.26		24w	7–14d
	ginkgo biloba extract	97	71	62.6		23.02 ± 4.68			

### Risk of bias, inconsistency, and heterogeneity

**Figure 2:**
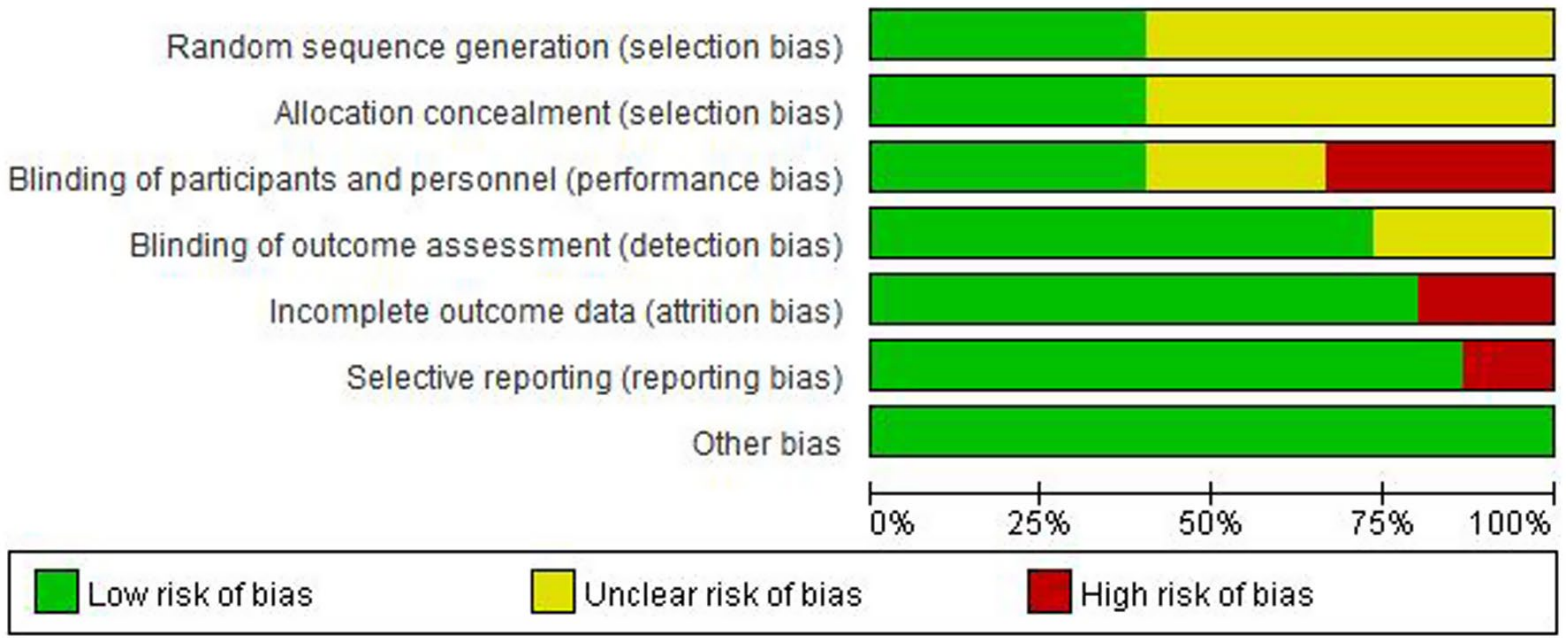
Risk of bias graph: review authors’ judgments about each risk of bias item presented as percentages across all included studies.

**Figure 3:**
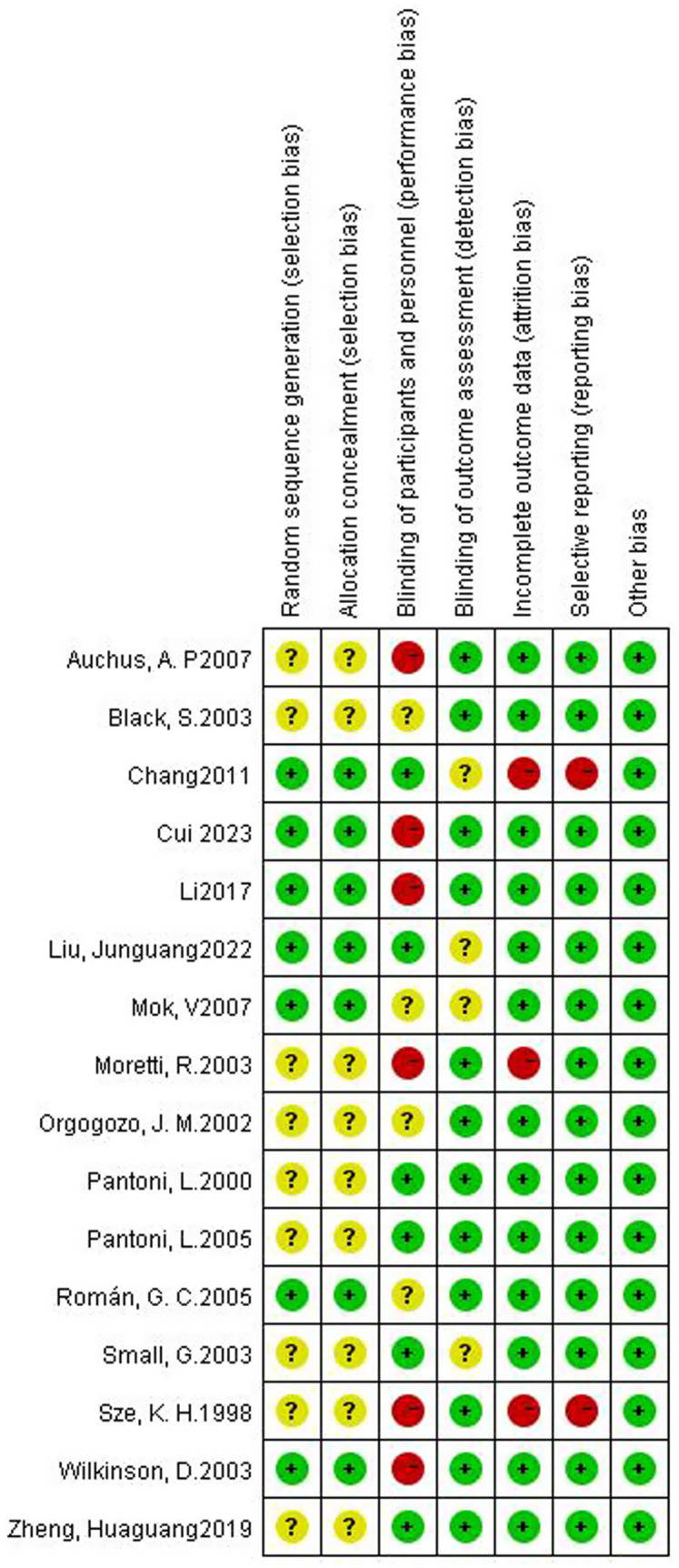
Risk of bias summary: review authors’ judgments about each risk of bias item for each included study.

For random sequence generation, 6 studies reported the use of a computer program-generated code randomization protocol, and 1 study reporting the use of a random permuted block design in which envelopes were sealed by persons not associated with the study were assigned a low risk of bias, and 9 studies not reporting how randomization was performed were assigned an unclear risk of bias. For Allocation concealment, there were 7 studies that met the criteria and were assigned a low risk of bias. For the blinding of participants and personnel, 6 trials that explicitly described double-blinding procedures and used placebos identical in appearance, dosage form, and dosage to the experimental drugs were rated as having a low risk of bias. 6 studies were assigned a high risk of bias due to the inability to be blinded. For the blinding of outcome assessment, 13 trials were assigned a low risk of bias. For Incomplete outcome data, 1 study was assigned a high risk of bias as reported severe cases dropped, and 2 studies that did not have adverse events data were assigned a high risk of bias. For selective reporting, 2 trials that did not have adverse event data were assigned a high risk of bias. For other biases, all trials were assigned a low risk of bias. Figures 2, 3 depicts the summary risk of bias for selected studies.

Since only the MMSE group included both direct and indirect comparisons (unlike the ADAS-cog and MoCA groups), we performed a global inconsistency test and a local inconsistency test for the MMSE group. The results for inconsistency were as follows: global inconsistency test, *p* = 0.771; local inconsistency assessment using the node-splitting model, *p* = 0.733 (Table 4). Therefore, we employed a consistency model for the network meta-analysis. Additionally, since the comparisons of nimodipine, rivastigmine, and placebo in the MMSE group formed a closed loop, we conducted a loop inconsistency test. The result indicated a loop-specific inconsistency value of 0, suggesting no major contradiction in this loop (Table 5).

**Table 4:** Assessment of inconsistency (Node-splitting model).

Nodes	Direct		Indirect		Difference		*p* value
	Coef.	SE	Coef.	SE	Coef.	SE	
Nimodipine-Placebo	0.13	0.16	0.80	1.96	−0.67	1.96	0.733
Rivastigmine-Placebo	0.70	1.88	0.03	0.57	0.67	1.96	0.733
Nimodipine-Rivastigmine	−0.10	0.55	0.57	1.89	−0.67	1.96	0.733

**Table 5:** Assessment of inconsistency (loop inconsistency).

Loop	IF	seIF	z_value	*p*_value	CI_95	Loop_Heterog_tau2
Pla-nim-riv	0.671	1.964	0.342	0.733	(0.00, 4.52)	0.00

Nim, nimodipine; Riv, rivastigmine; Pla, placebo.

**Figure 4:**
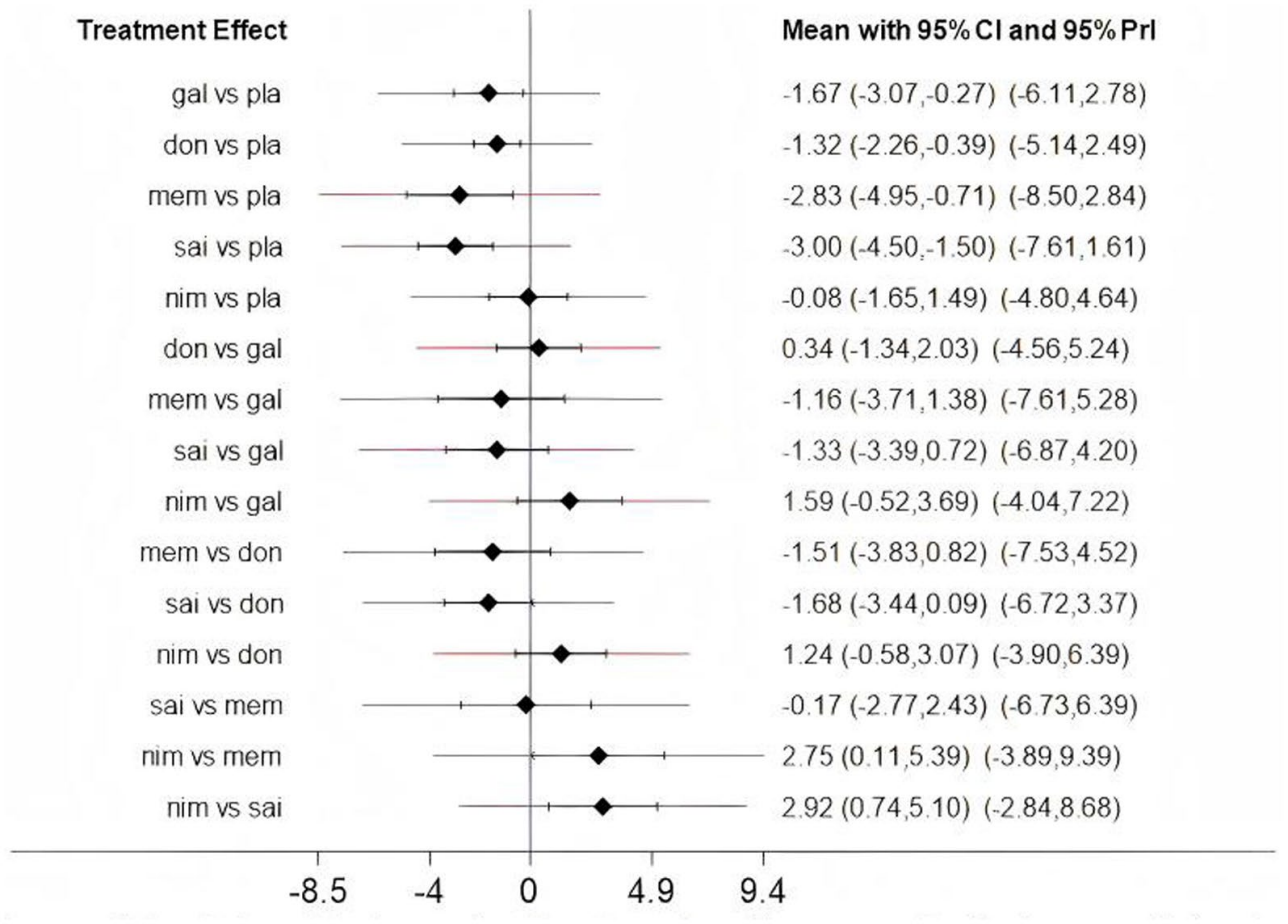
The predictive interval between fixed and random (Assessment of heterogeneity); gal, galantamine; don, donepezil; mem, memantine; sai, sailuotong; nim, nimodipine; pla, placebo.

**Figure 5:**
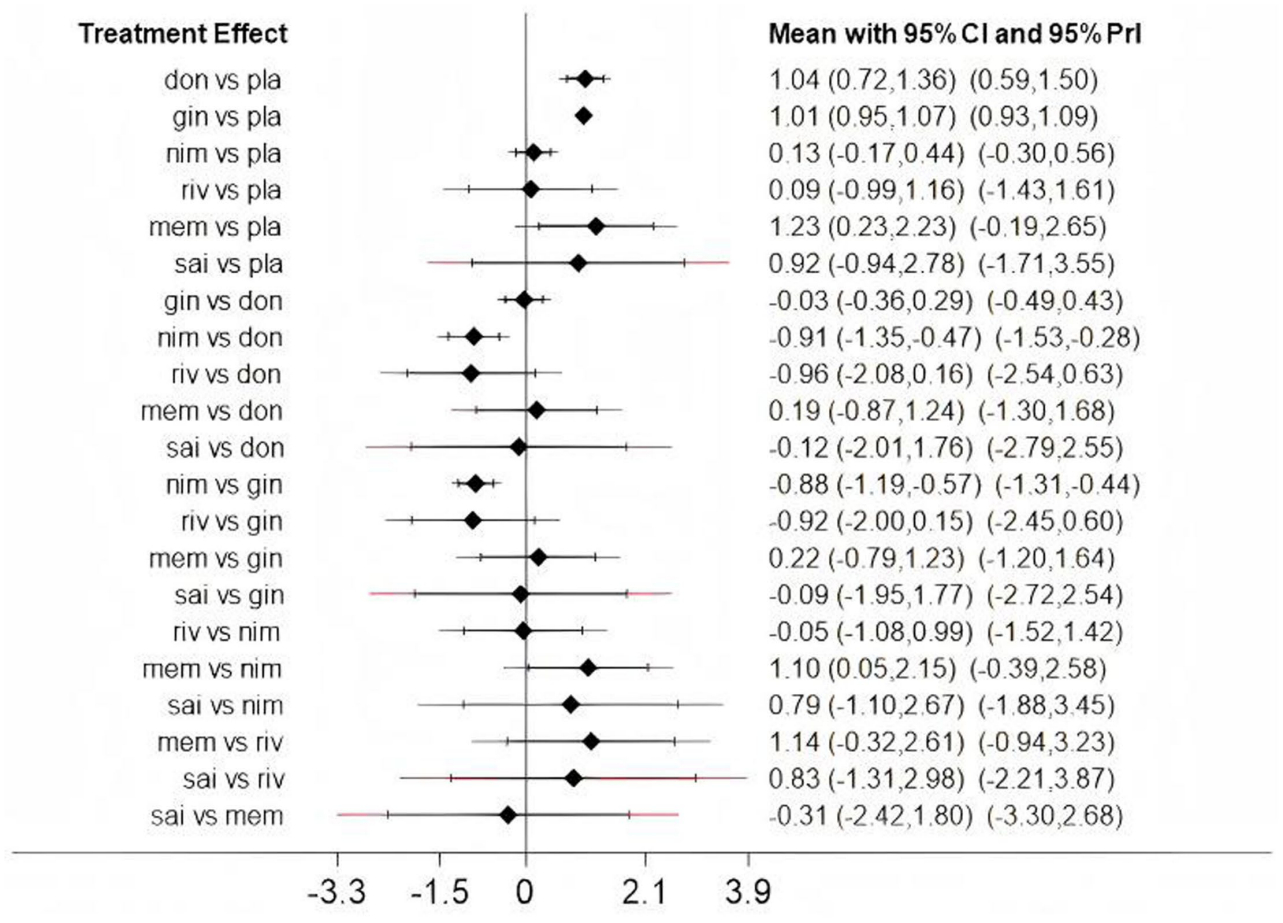
The predictive interval between fixed and random (Assessment of heterogeneity); don, donepezil; gin, ginkgo biloba extract; nim, nimodipine; riv, rivastigmine; mem, memantine; sai, sailuotong; pla, placebo.

Low heterogeneity was found across several comparisons for both outcomes, as measured by the prediction interval [Figure 4 (ADAS-cog), Figure 5 (MMSE)].

### Pairwise meta-analysis

**Figure 6:**
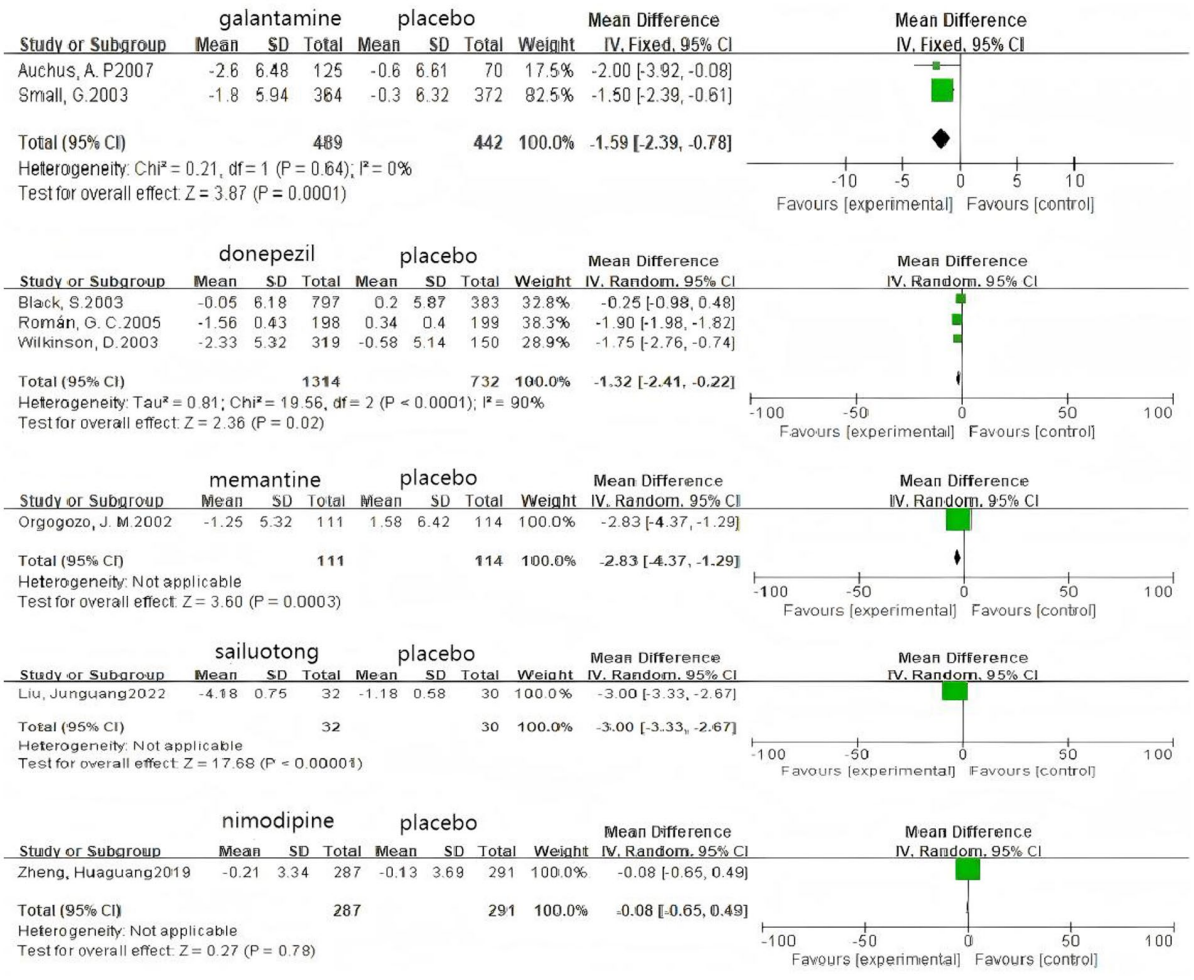
Forest plots of the pair wise meta-analysis of ADAs-cog.

**Figure 7:**
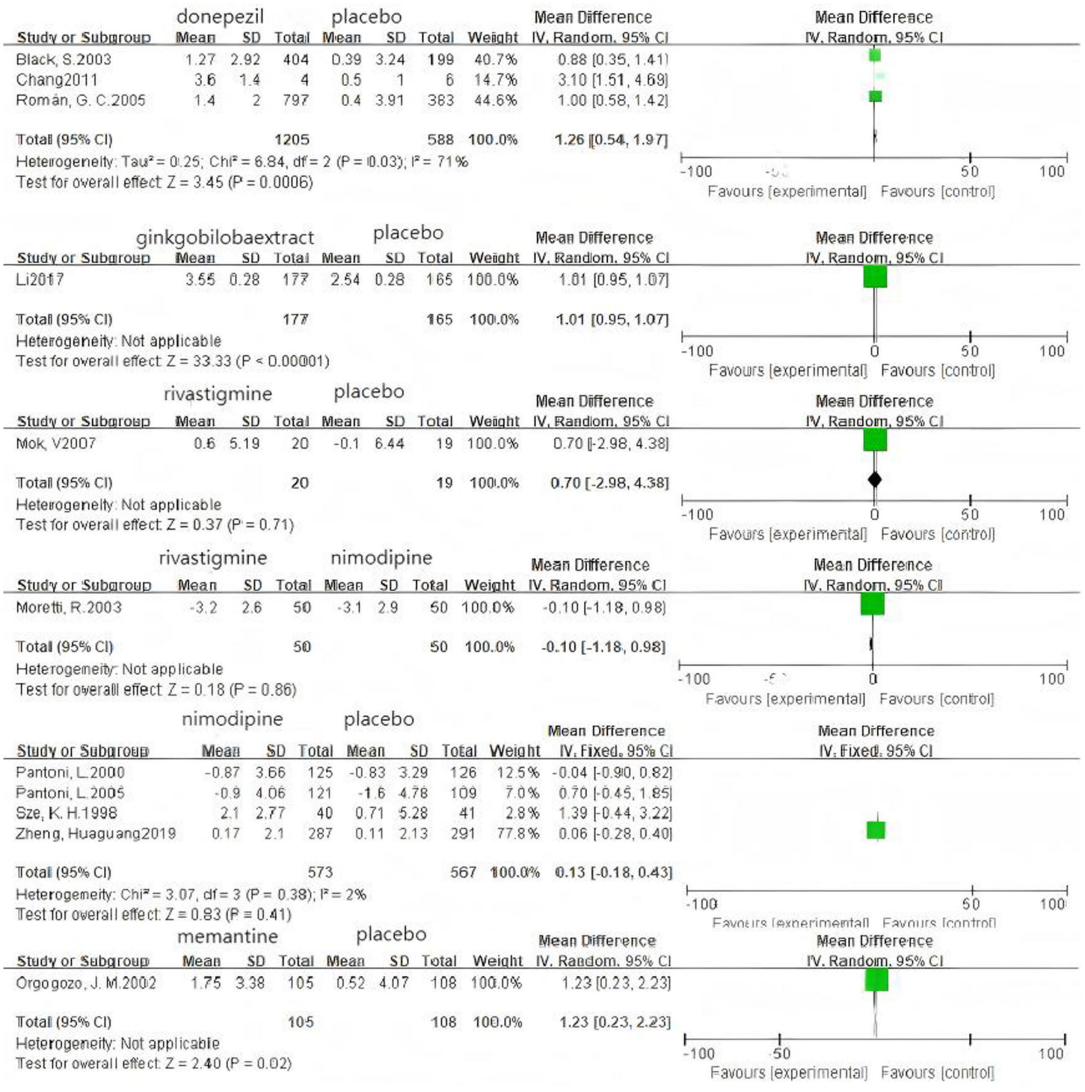
Forest plots of the pairwise meta-analysis of MMSE.

**Figure 8:**
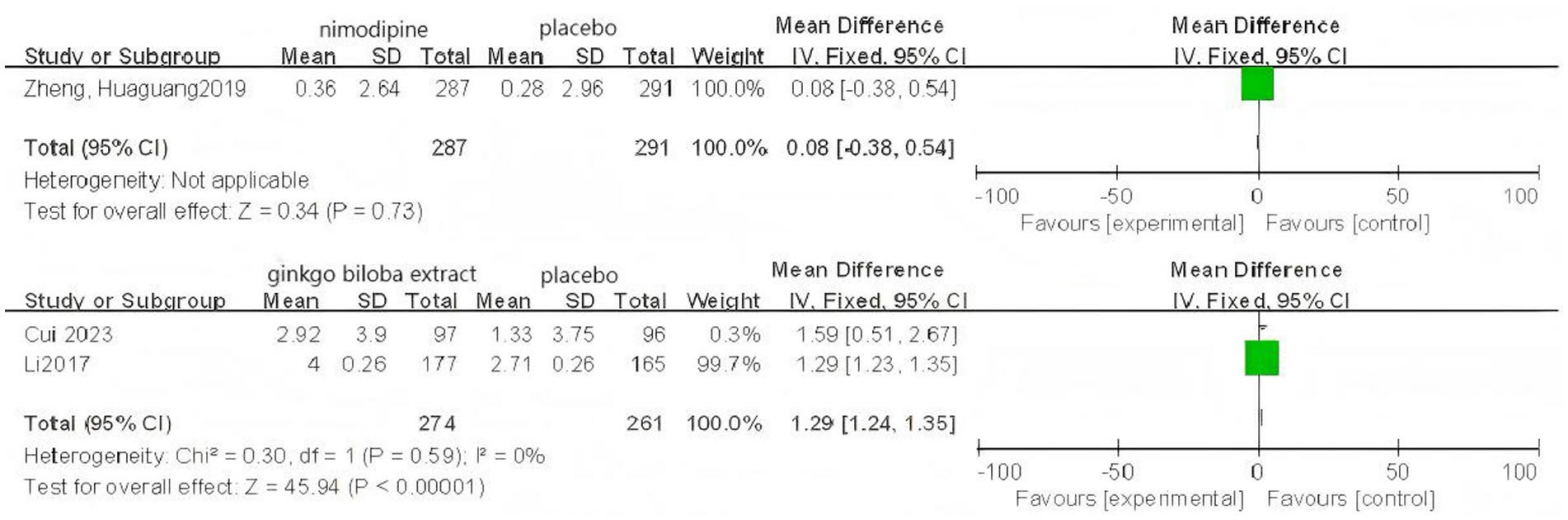
Forest plots of the pairwise meta-analysis of MoCA.

Based on 3 cognitive assessment scales. 8 direct pairwise meta-analyses were conducted to compare the ADAS-cog score (Figure 6), 11 to compare the MMSE score (Figure 7), and 3 to compare the MoCA score (Figure 8), respectively, which can be summarily seen in Table 6. As for the ADAS-cog outcome, galantamine (MD = −1.59, 95% CI: −2.39, −0.78), donepezil (MD = −1.32, 95% CI: −2.41, −0.22), memantine (MD = −2.83, 95% CI: −4.37, −1.29), sailuotong (MD = −3.00, 95% CI: −3.33, −2.67) and nimodipine (MD = −0.08, 95% CI: −0.65, 0.49) were more efficient than placebo, however, there was no statistical difference in efficacy between nimodipine and placebo. As for the MMSE outcome, donepezil (MD = 1.26, 95% CI: 0.54, 1.97), ginkgo biloba extract (MD = 1.01, 95% CI: 0.95, 1.07), rivastigmine (MD = 0.70, 95% CI: −2.98, 4.38), nimodipine (MD = 0.13, 95% CI: −0.18, 0.43) and memantine (MD = 1.23, 95% CI: 0.23, 2.23) were more efficient than placebo, nimodipine (MD = −0.10, 95% CI: −1.18, 0.98) is more efficient than rivastigmine. The sparse network geometry for MoCA led to a pairwise meta-analysis, which showed that ginkgo biloba extract (MD = 1.29, 95% CI: 1.24, 1.35) and nimodipine (MD = 0.08, 95% CI: −0.38, 0.54) were more effective than placebo.

**Table 6:** Pairwise meta-analysis.

Comparison	MD (95%CI)	Number of patients	Number of studies	Heterogeneity test	
				*I* ^2^	*p*-value
ADAS-cog					
Gal-Pla	−1.59 (−2.39, −0.78)	931	2	0%	0.64
Don-Pla	−1.32 (−2.41, −0.22)	2046	3	90%	<0.0001
Mem-Pla	−2.83 (−4.37, −1.29)	225	1	–	–
Sai-Pla	−3.00 (−3.33, −2.67)	62	1	–	–
Nim-Pla	−0.08 (−0.65, 0.49)	578	1	–	–
MMSE					
Don-Pla	1.26 (0.54, 1.97)	1793	3	71%	0.03
Gin-Pla	1.01 (0.95, 1.07)	342	1	–	–
Riv-Pla	0.70 (−2.98, 4.38)	39	1	–	–
Riv-Nim	−0.10 (−1.18, 0.98)	100	1	–	–
Nim-Pla	0.13 (−0.18, 0.43)	1,140	4	2%	0.38
Mem-Pla	1.23 (0.23, 2.23)	213	1	–	–
MoCA					
Gin-Pla	1.29 (1.24, 1.35)	535	2	0%	0.59
Nim-Pla	0.08 (−0.38, 0.54)	578	1	–	–

Pla, placebo; Gal, galantamine; Don, donepezil; Mem, memantine; Sai, sailuotong; Nim, nimodipine; Riv, rivastigmine; Gin, ginkgo biloba extract.

### Network meta-analysis

**Figure 9:**
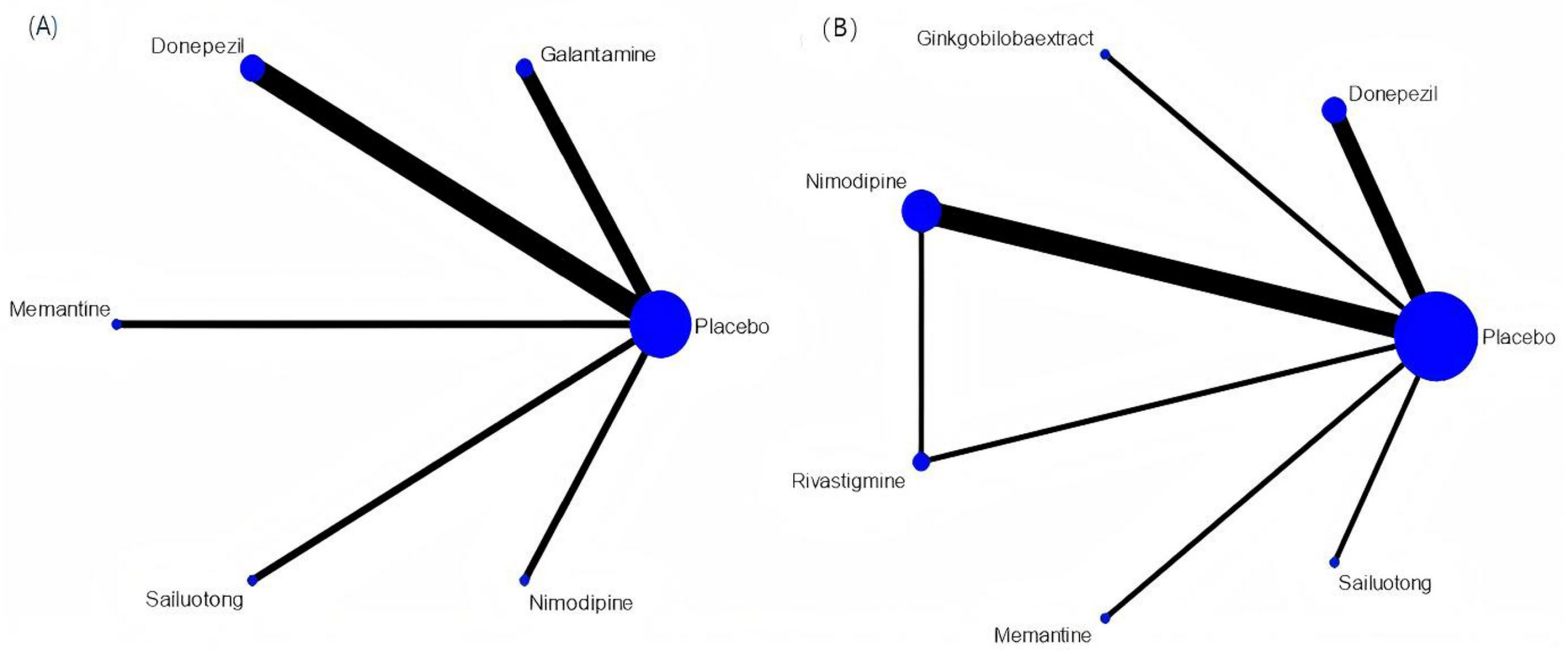
Networkmap. **(A)** ADAS-cog, **(B)** MMSE. Width of the lines is proportional to the number of trial. Size of every circle is proportional to the number of randomly assigned participants (sample size).

**Figure 10:**
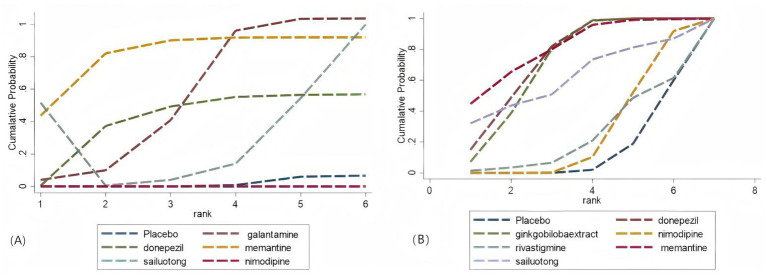
Cumulative probability ranking curve. **(A)** ADAS-cog; **(B)** MMSE. The vertical axis represents cumulative probabilities, while the horizontal axis represents ranks.

Network meta-analysis was performed using StataSE 16.0 with the network package, generating network maps (Figures 9A,B) and the cumulative ranking curve based on ADAS-cog and MMSE (Figures 10A,B), along with a league table (Table 7). For ADAS-cog (8 studies, 3,689 participants), pooled data showed that sailuotong (MD = −3.00, 95% CI: −4.50, −1.50), memantine (MD = −2.83, 95% CI: −4.95, −0.71), galantamine (MD = −1.67, 95% CI: −3.07, −0.27), and donepezil (MD = −1.32, 95% CI: −2.26, −0.39) were significantly more beneficial than placebo, with sailuotong and memantine outperforming nimodipine. Therapy ranking by efficacy was sailuotong>memantine>galantamine>donepezil>nimodipine>placebo, where sailuotong had the highest SUCRA value (88.5%), followed by memantine (83.1%) (Table 8). For MMSE (8 studies, 3,842 participants), pooled data indicated memantine (MD = 1.23, 95% CI: 0.23, 2.23), donepezil (MD = 1.04, 95% CI: 0.72, 1.36), and ginkgo biloba extract (MD = 1.01, 95% CI:0.95, 1.07) were superior to placebo, with memantine, donepezil, and ginkgo biloba extract outperforming nimodipine. Therapy ranking by efficacy was memantine>donepezil>ginkgo biloba extract>sailuotong>nimodipine>rivastigmine>placebo, where memantine showed the highest SUCRA value (80.8%) (Table 9).

**Table 7:** League table.

ADAS-cog					
Sailuotong					
−0.17 (−2.77, 2.43)	Memantine				
−1.33 (−3.39, 0.72)	−1.16 (−3.71, 1.38)	Galantamine			
−1.68 (−3.44, 0.09)	−1.51 (−3.83, 0.82)	−0.34 (−2.03, 1.34)	Donepezil		
−2.92 (−5.10, −0.74)	−2.75 (−5.39, −0.11)	−1.59 (−3.69, 0.52)	−1.24 (−3.07, 0.58)	Nimodipine	
−3.00 (−4.50, −1.50)	−2.83 (−4.95, −0.71)	−1.67 (−3.07, −0.27)	−1.32 (−2.26, −0.39)	−0.08 (−1.65, 1.49)	Placebo

MMSE						
Memantine						
0.19 (−0.87, 1.24)	Donepezil					
0.22 (−0.79, 1.23)	0.03 (−0.29, 0.36)	*Ginkgo biloba* extract				
0.31 (−1.80, 2.42)	0.12 (−1.76, 2.01)	0.09 (−1.77, 1.95)	Sailuotong			
1.10 (0.05, 2.15)	0.91 (0.47, 1.35)	0.88 (0.57, 1.19)	0.79 (−1.10, 2.67)	Nimodipine		
1.14 (−0.32, 2.61)	0.96 (−0.16, 2.08)	0.92 (−0.15, 2.00)	0.83 (−1.31, 2.98)	0.05 (−0.99, 1.08)	Rivastigmine	
1.23 (0.23, 2.23)	1.04 (0.72, 1.36)	1.01 (0.95, 1.07)	0.92 (−0.94, 2.78)	0.13 (−0.17, 0.44)	0.09 (−0.99, 1.16)	Placebo

**Table 8:** The SUCRA value of MMSE.

Treatment	SUCRA
Donepezil	74.0
*Ginkgo biloba* extract	70.8
Nimodipine	25.8
Rivastigmine	23.8
Memantine	80.8
Sailuotong	61.4

**Table 9:** The SUCRA value of ADAS-cog.

Treatment	SUCRA
Galantamine	57.6
Donepezil	47.3
Memantine	82.8
Sailuotong	88.2
Nimodipine	14.4

**Figure 11:**
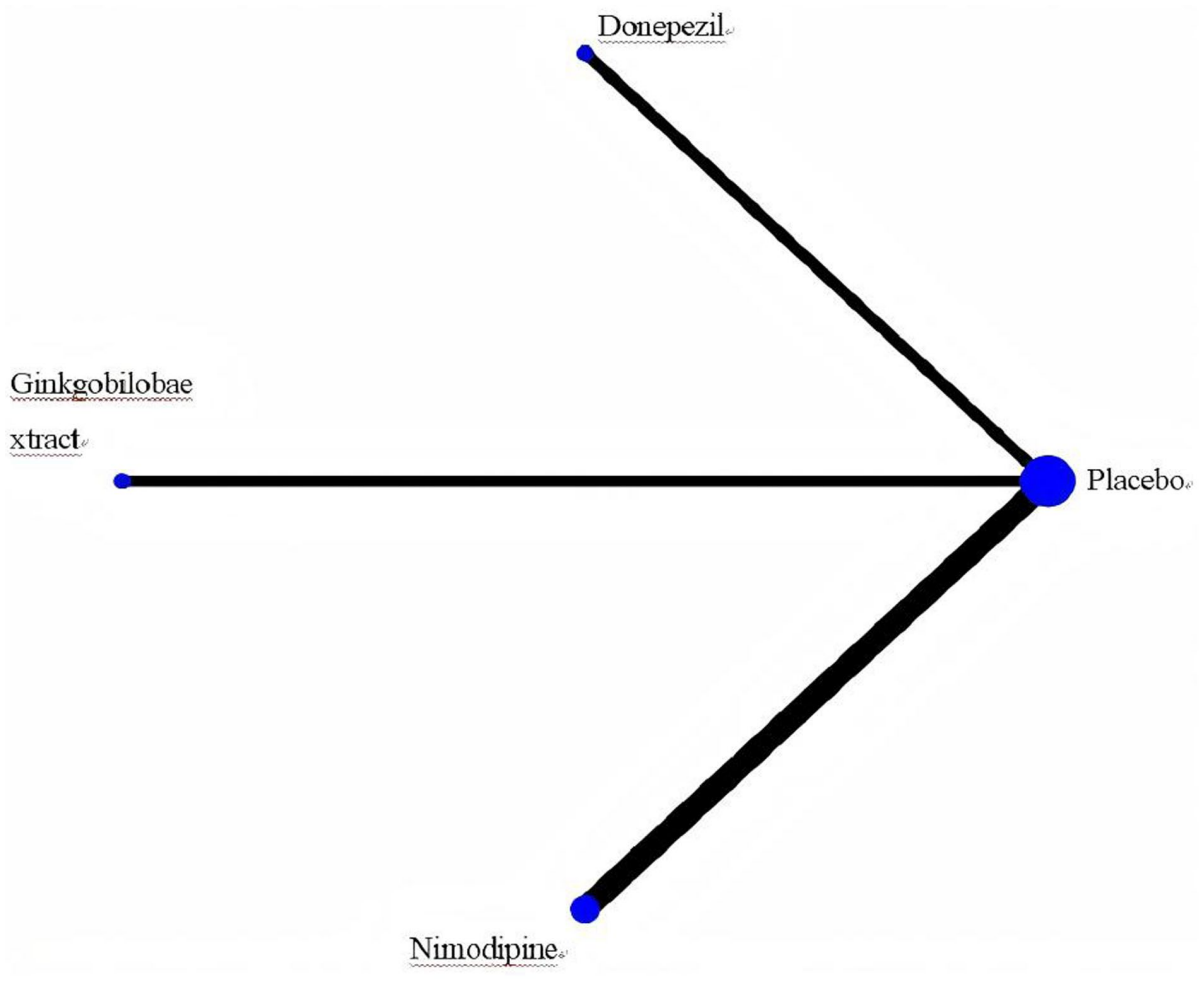
Network map of PSCl. Width of the lines is proportional to the number of trial. Size of every circle is Proportional to the number of randomly assigned participants (sample size).

**Figure 12:**
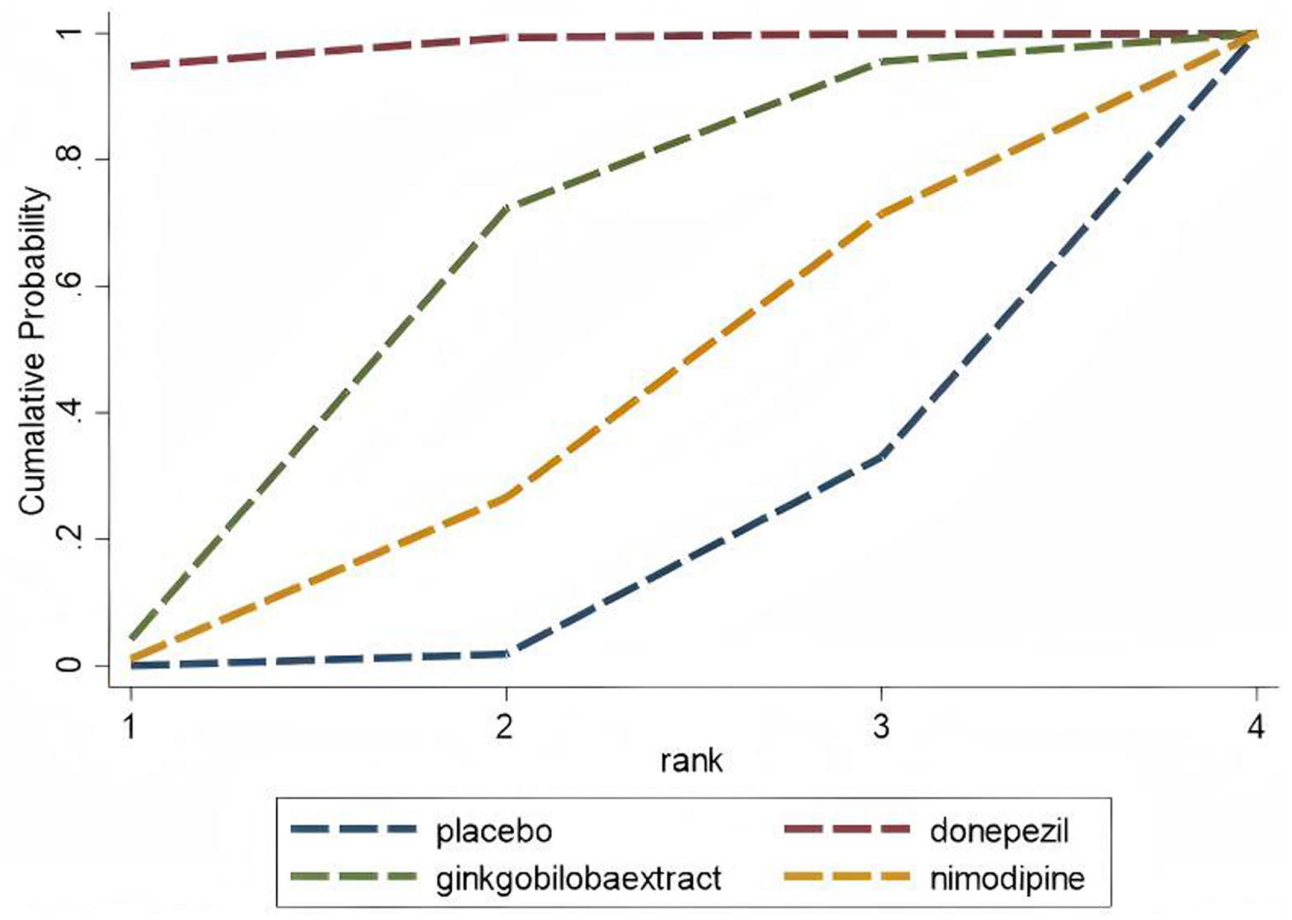
Cumulative probability ranking curve of PSCl The vertical axis represents cumulative probabilities, while the horizontal axis represents ranks.

A separate analysis of PSCI as an important subtype of VCI showed the following results: For the MMSE scale (5 studies, 1,309 participants), the network map is shown in Figure 11, along with a league table (Table 10). Pooled data showed that donepezil (MD = 2.69, 95% CI: 0.19, 5.19) was significantly more beneficial than nimodipine. Therapy ranking by efficacy was donepezil > ginkgo biloba extract > nimodipine > placebo, where donepezil had the highest SUCRA value (98%). The cumulative ranking curve is shown in Figure 12. Therapy ranking by efficacy was donepezil>ginkgo biloba extract>nimodipine>placebo (Table 11). For the ADAS-cog scale, only one study was available, and for the MoCA scale, two studies were available; therefore, a pairwise meta-analysis was conducted, with results shown in Figure 8.

**Table 10:** League table of PSCI.

Donepezil			
2.09 (−0.33, 4.51)	*Ginkgo biloba* extract		
2.69 (0.19, 5.19)	0.60 (−1.34, 2.54)	Nimodipine	
3.10 (1.05, 5.15)	1.01 (−0.28, 2.30)	0.41 (−1.03, 1.85)	Placebo

**Table 11:** The SUCRA value of ADAS-cog.

Treatment	SUCRA
Donepezil	98.0
*Ginkgo biloba* extract	57.3
Nimodipine	33.0

### Safety assessment

**Figure 13:**
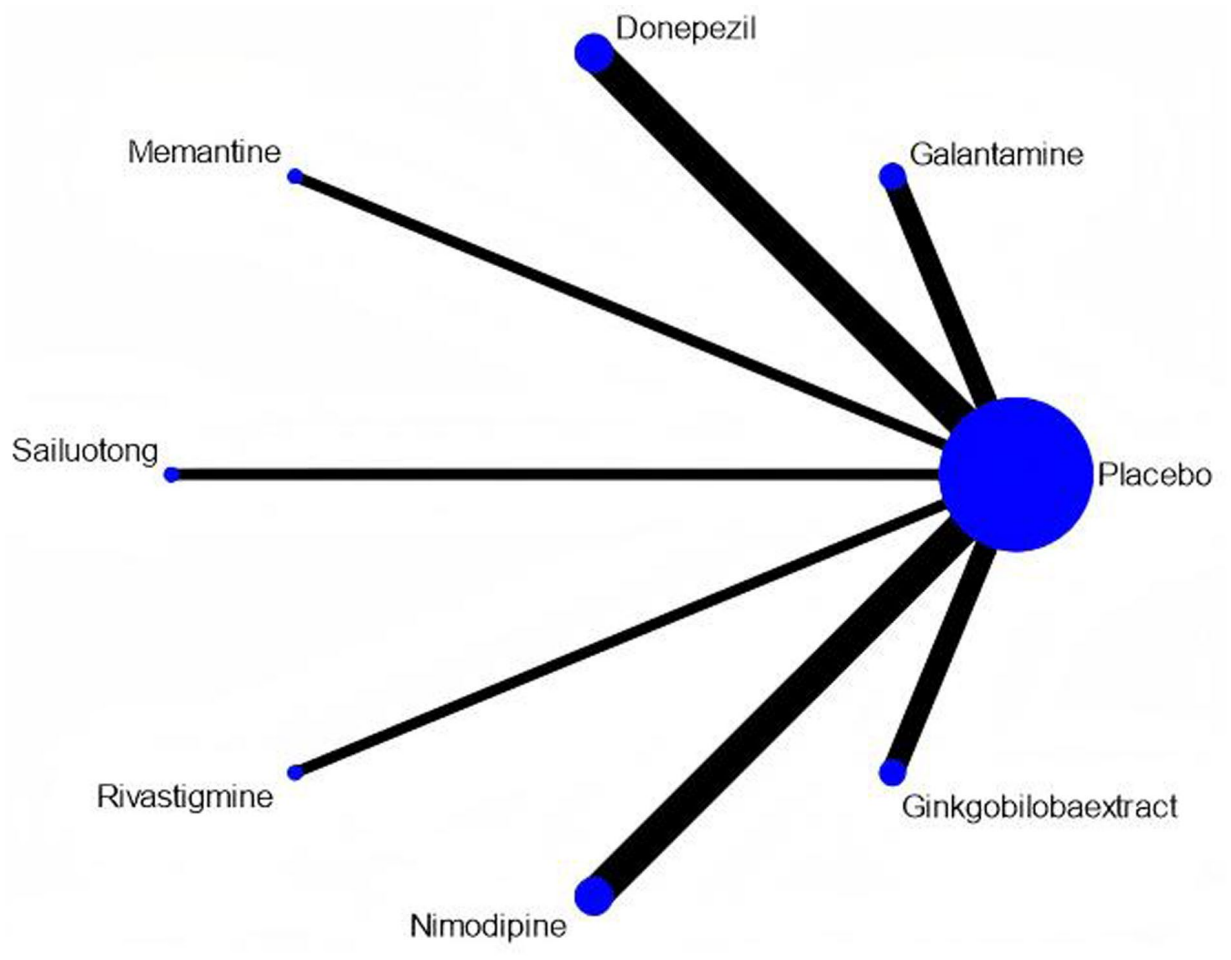
Network map of adverse events. Width of the lines is proportional to the number of trial. Size of Every circle is proportional to the number of randomly assigned participants (sample size).

**Figure 14:**
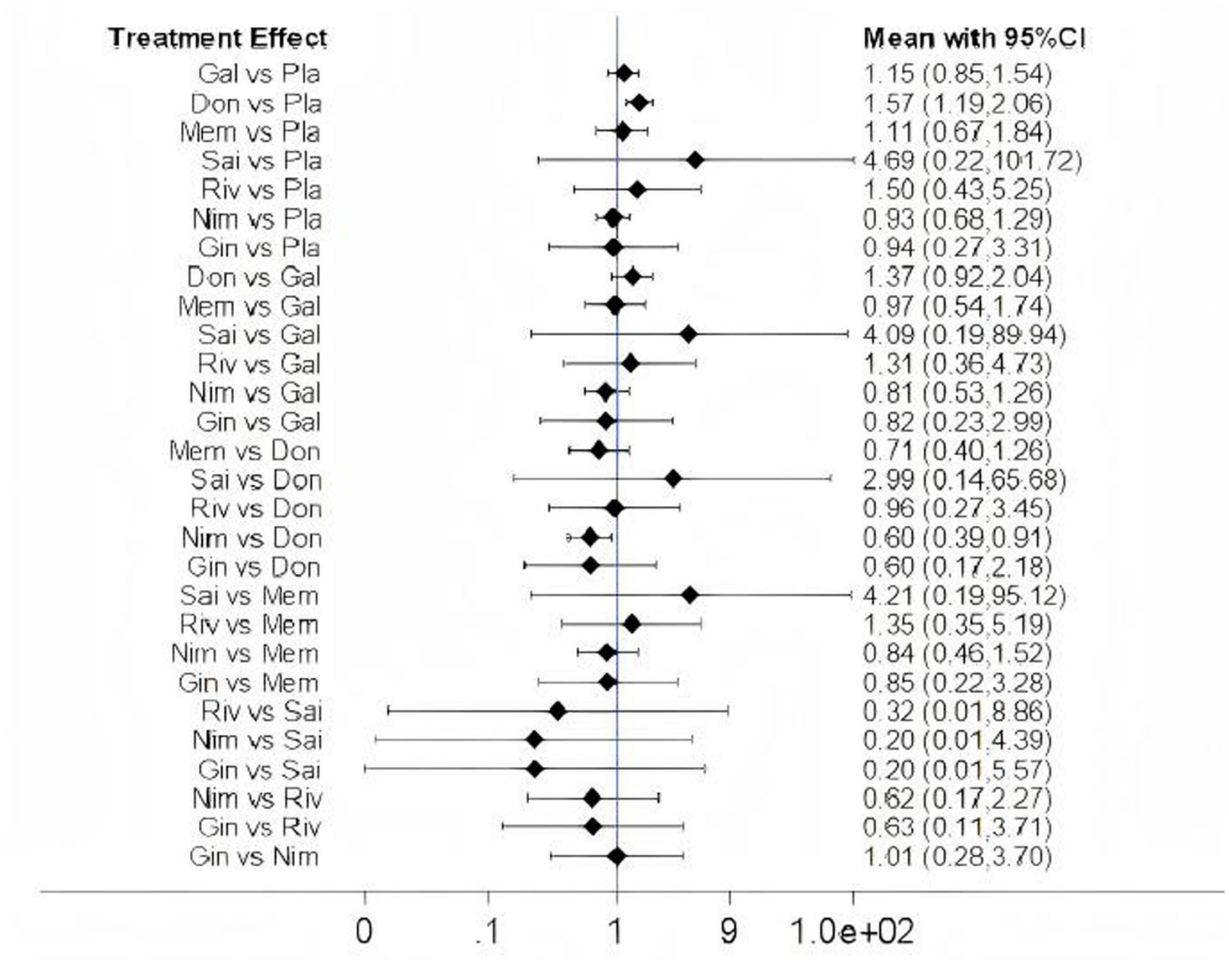
Forest plots of adverse events. Gal, galantamine. Don, donepezil. Mem, memantine. Sai, sailuotong. Riv, rivastigmine. Nim, nimodipine. Gin, ginkgo biloba extract. Pla, placebo.

Among the 15 included studies, 13 studies reported data on adverse events associated with cognitive-enhancing medications, while 2 studies (one on donepezil and one on nimodipine) lacked adverse event data. One rivastigmine study reported no adverse events. The network map, forest plot, and league table are presented in Figures 13, 14, Table 12. Network meta-analysis revealed that donepezil (OR: 1.57; 95% CI: 1.19–2.06) significantly increased the risk of overall adverse events compared to placebo. No statistically significant differences in adverse event rates were observed for galantamine, memantine, sailuotong, rivastigmine, nimodipine, or ginkgo biloba extract relative to placebo.

**Table 12:** League table of adverse events.

Sailuotong							
1.10 (−1.99, 4.18)	Donepezil						
1.14 (−2.18, 4.46)	0.04 (−1.24, 1.33)	Rivastigmine					
1.41 (−1.68, 4.50)	0.31 (−0.09, 0.71)	0.27 (−1.02, 1.55)	Galantamine				
1.44 (−1.68, 4.56)	0.34 (−0.23, 0.91)	0.30 (−1.05, 1.65)	0.03 (−0.55, 0.61)	Memantine			
1.61 (−1.72, 4.93)	0.51 (−0.78, 1.80)	0.47 (−1.31, 2.24)	0.20 (−1.10, 1.49)	0.17 (−1.19, 1.52)	*Ginkgo biloba* extract		
1.55 (−1.53, 4.62)	0.45 (0.18, 0.72)	0.41 (−0.85, 1.66)	0.14 (−0.16, 0.43)	0.11 (−0.40, 0.61)	−0.06 (−1.32, 1.20)	Placebo	
1.61 (−1.48, 4.71)	0.52 (0.10, 0.94)	0.47 (−0.82, 1.77)	0.21 (−0.23, 0.64)	0.18 (−0.42, 0.77)	0.01 (−1.29, 1.31)	0.07 (−0.25, 0.39)	Nimodipine

### Publication bias

**Figure 15:**
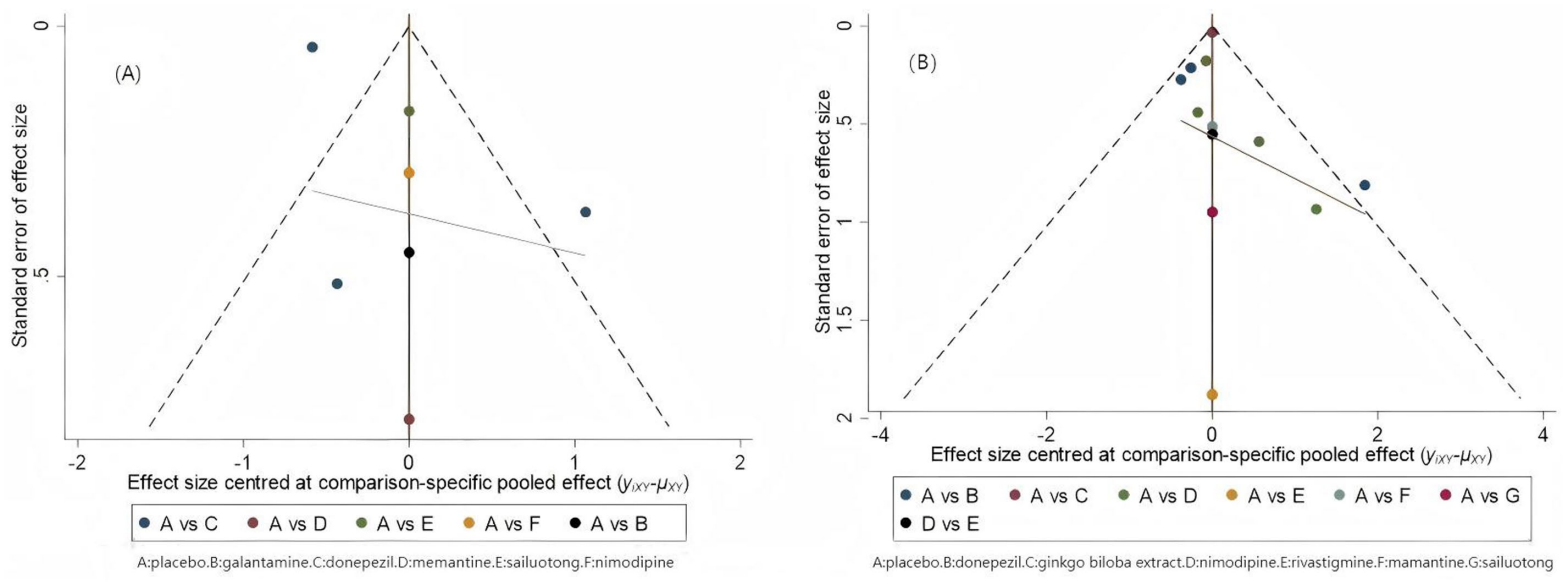
Comparison-adjusted funnelplots. **(A)** ADAS-cog; A, placebo; B, galantamine; C, donepezil; D, memantine; E, sailuotong; F, nimodipine. **(B)** MMSE; A, placebo, B, donepezil, C, ginkgo biloba extract, D, nimodipine, E, rivastigmine, F, memantine, G, sailuotong.

Funnel plots were performed to evaluate publication bias and small-study effects for ADAS-cog and MMSE, respectively. Visual inspection of the funnel plots indicated approximate symmetry for both ADAS-cog and MMSE assessments, suggesting a low risk of publication bias (Figures 15A,B).

### GRADE evaluation on the quality of evidence

According to GRADE, the quality of the evidence is in the range of very low and low. In terms of donepezil vs. placebo, the quality was very low for ADAS-cog, low for MMSE. As for ginkgo biloba extract vs. placebo and rivastigmine vs. placebo, the quality was low for MMSE. The details are shown in Table 13.

**Table 13:** GRADE framework.

Comparison	Risk of bias	Inconsistency	Indirectness	Imprecision	Publication bias	GRADE
ADAS-cog						
Gal vs. Pla	Serious	Not serious	Serious	Not serious	Not serious	Low
Don vs. Pla	Very serious	Very serious	Serious	Not serious	Serious	Very low
Mem vs. Pla	Not serious	Not serious	Serious	Serious	Not serious	Low
Sai vs. Pla	Not serious	Not serious	Serious	Serious	Not serious	Low
Nim vs. Pla	Not serious	Not serious	Not serious	Very serious	Not serious	Low
MMSE						
Don vs. Pla	Not serious	Serious	Serious	Not serious	Not serious	Low
Gin vs. Pla	Serious	Not serious	Not serious	Serious	Not serious	Low
Riv vs. Pla	Not serious	Not serious	Serious	Very serious	Not serious	Very low
Nim vs. Riv	Very serious	Not serious	Not serious	Very serious	Not serious	Very low
Nim vs. Pla	Serious	Not serious	Serious	Serious	Serious	Low
Mem vs. Pla	Not serious	Not serious	Serious	Serious	Not serious	Low
MoCA						
Gin vs. Pla	Not serious	Not serious	Not serious	Serious	Not serious	Moderate

Pla, placebo; Gal, galantamine; Don, donepezil; Mem, memantine; Sai, sailuotong; Nim, nimodipine; Riv, rivastigmine; Gin, ginkgo biloba extract.

## Discussion

This network meta-analysis compared the efficacy and safety profiles of cognitive-enhancing drugs for VCI and PSCI. Regarding ADAS-cog score reduction, sailuotong showed the most significant effect (MD = −3.00, 95% CI: −4.50, −1.50) with the highest SUCRA value (88.5%), followed by memantine (MD = −2.83, 95% CI: −4.95, −0.71) with SUCRA value 83.1%. For MMSE improvement, memantine showed the most significant effect (MD = 1.23, 95% CI: 0.23, 2.23), followed by donepezil (MD = 1.04, 95% CI: 0.72, 1.36), and ginkgo biloba extract (MD = 1.01, 95% CI: 0.95, 1.07). Safety analysis revealed that donepezil significantly increased adverse event risk (OR: 1.57, 95% CI: 1.19, 2.06) versus placebo. Considering both efficacy and safety profiles, memantine emerges as the optimal pharmacological choice for PSCI, followed by sailuotong and donepezil, this is in line with the latest VCI guidelines (3), which recommend cholinesterase inhibitors (such as donepezil, galantamine, and rivastigmine) and N-methyl-D-aspartate receptor antagonists (such as memantine) as first-line medications for VCI.

Memantine is an uncompetitive NMDA receptor antagonist at therapeutic concentrations achieved in the treatment of dementia and is essentially devoid of such side effects at doses within the therapeutic range (11). Multiple clinical studies have demonstrated the efficacy of memantine in enhancing cognitive function and activities of daily living among patients with diverse etiologies of cognitive impairment (12–15).

Donepezil is a reversible acetylcholinesterase inhibitor. Existing studies have demonstrated that acetylcholinesterase inhibitors can improve cognitive function in patients with VCI by inhibiting the hydrolysis of acetylcholine in the brain (16). In clinical practice, donepezil is primarily used to improve cognition, global functioning, and activities of daily living in patients with mild-to-moderate AD; however, it only shows a mild effect in PSCI patients and has relatively frequent side effects, making it unsuitable for widespread clinical use (17, 18).

Sailutong is a complex herbal formulation that consists of standardized extracts of *Ginkgo biloba* (Ginkgo), Panax ginseng (Ginseng), and Crocus sativa (Saffron), which have been suggested to have antioxidant, anti-inflammatory, and anti-apoptotic effects, slowing down the cognitive impairment process in subjects with VCI (19), however, research on traditional Chinese medicine for PSCI has rarely been published in international journals outside of China and has not gained widespread global recognition. Sailuotong is acknowledged in Chinese medicine for improving blood flow and cognitive function. However the clinical evidence often comes from small, poorly designed studies. So, future validation through high-quality research is essential.

Furthermore, this study has limitations, including a potential language bias as the inclusion of studies was restricted to those published in English and Chinese, which may limit the generalizability of the findings. Among the ten studies investigating VCI, the majority applied the National Institute of Neurological Disorders and Stroke–Association Internationale pour la Recherche et l’Enseignement en Neurosciences (NINDS-AIREN) criteria, which contributed to reduced heterogeneity across these studies. For the six studies focusing specifically on PSCI, the time post-stroke at which participants were enrolled varied. Current PSCI management guidelines identify the 3–6 month period post-stroke as the optimal intervention window, noting that interventions initiated beyond 6 months are associated with poorer long-term cognitive outcomes (5). In the present analysis, all but one of the included PSCI studies defined the intervention timeframe as within 6 months post-stroke. One study specified enrolment at >3 months, making its classification into ≤6 or >6 months ambiguous; consequently, the variation in timing likely had a minimal impact on the comparative drug efficacy results.

Substantial variability was observed in treatment durations across the 16 included studies. Nimodipine was investigated over the widest range (12 to 52 weeks), yet demonstrated consistently limited cognitive benefit. In contrast, Sailuotong, administered for only 16 weeks, was associated with the second-highest ranked efficacy among all interventions. However, the mechanisms of action and efficacy of Sailuotong are unclear compared to drugs like memantine, donepezil, nimodipine, and rivastigmine. Furthermore, this finding is based on a single study with a small sample size (*n* = 62), and comparative estimates involving Sailuotong may be unstable due to network sparsity. Other pharmacotherapies—including galantamine, donepezil, rivastigmine, and memantine—were administered over consistent periods (24–26 weeks), suggesting that treatment duration was unlikely to be a major source of efficacy variation for these agents.

An important methodological consideration is the predominant use of cognitive assessment scales originally developed for AD (e.g., ADAS-cog), rather than instruments specifically validated for vascular cognitive impairment. This may introduce measurement bias in estimating treatment effects. Future trials in VCI should prioritize the use of vascular-specific cognitive measures.

Furthermore, stratification by cognitive severity and vascular pathogenesis (e.g., strategic, cortical, or subcortical lesions) is warranted, as treatment efficacy may differ across these subgroups (18). Among the included studies, cognitive severity varied considerably: the cohort in Mok et al. (20) had the highest severity, followed by Orgogozo et al. (15), while the mildest impairment was reported in Zheng et al. (21), followed by Sze et al. (22) and Chang et al. (23). These differences likely contributed to clinical heterogeneity. Regarding vascular pathogenesis, the memantine trial which showed favourable outcomes, included patients with large cortico-subcortical lesions, white-matter changes, and circumscribed subcortical/lacunar lesions. The two donepezil trials by Román et al. (24) and Black et al. (25) also enrolled populations with predominantly subcortical pathology and reported positive results. Conversely, neither rivastigmine (20) nor nimodipine (26) demonstrated significant efficacy in cohorts with subcortical lesions, and a nimodipine trial in multi-infarct dementia (27) also showed limited benefit. Vascular pathogenesis was not clearly detailed in the remaining studies, limiting subtype-specific interpretations.

Our network meta-analysis suggests that memantine might be the most effective for PSCI, followed by sailuotong, but these findings are preliminary. As the estimates are based on a small randomized controlled trial and a sparse network, the evidence remains limited and requires confirmation through future high-quality studies.

## Conclusion

The results of this study provide clinically relevant evidence for improving prognosis in VCI and PSCI patients, demonstrating that pharmacological interventions exert measurable positive effects on cognitive function in this population. Based on our analysis, memantine emerges as the most promising first-line treatment for PSCI-related cognitive impairment, followed by sailuotong and donepezil as secondary options. Nimodipine showed limited cognitive benefits, though further studies are warranted to confirm these therapeutic hierarchies.

## Data Availability

The data analyzed in this study is subject to the following licenses/restrictions: data sharing is not applicable to this article as no new data were created or analyzed in this study. Requests to access these datasets should be directed to wenting li, 13438674841@163.com.
